# Adverse childhood experiences and health outcomes: a 20-year real-world study

**DOI:** 10.3389/fmed.2024.1429137

**Published:** 2025-01-07

**Authors:** Bárbara Martins, Tiago Taveira-Gomes, Joana Costa Gomes, Maria João Vidal-Alves, Teresa Magalhães

**Affiliations:** ^1^Department of Public Health and Forensic Sciences, and Medical Education, Faculty of Medicine, University of Porto, Porto, Portugal; ^2^Center for Health Technology and Services Research (CINTESIS@RISE), Faculty of Medicine, University of Porto, Porto, Portugal; ^3^Faculty of Health Sciences, University Fernando Pessoa (FCS-UFP), Porto, Portugal; ^4^SIGIL Scientific Enterprises, Dubai, United Arab Emirates; ^5^Local Healthcare Unit of Matosinhos, Senhora da Hora, Portugal; ^6^Epidemiology Unit (EPIUnit), Laboratory for Integrative and Translational Research in Population Health (ITR), Porto, Portugal; ^7^TOXRUN–Toxicology Research Unit, University Institute of Health Sciences, Advanced Polytechnic and University Cooperative (CESPU), CRL, Gandra, Portugal

**Keywords:** adverse childhood experiences, suspected victims, health outcomes, traumatic injuries, intoxications, mental health disorders, physical disorders

## Abstract

**Introduction:**

Adverse childhood experiences (ACEs) refer to traumatic life events occurred in childhood that comprise abuse (e.g., psychological, physical, sexual), neglect (psychological and physical), indirect violence or household dysfunctions. Such experiences ultimately lead to severe short-, medium- and long-term consequences for the victim’s health. The goal of this study is to analyze the prevalence of health outcomes in children <16 years of age, who were suspected of ACEs by physicians. The specific objectives consist of analyzing 3 health outcome groups: (a) traumatic injuries and intoxications; (b) mental health disorders; and (c) physical disorders.

**Methods:**

We performed a real-world, retrospective, observational, cross-sectional, and multicentric study, using complementary data from electronic health records and healthcare registries from the local healthcare unit of Matosinhos, generated between January 1, 2001, and December 31, 2021 (total child population <16 years observed during that period=40 536). Keywords and ICD-9, ICD-10, and ICPC-2 codes were applied to find data on the victims.

**Results:**

Just over 2% of children were referred to as victims in the available information (n=918). Social problems, injuries and intoxications, mental health disorders, and physical disorders were observed at higher percentages in suspected victims than in the total analyzed population.

**Discussion:**

These results reveal that child victims of ACEs may be underdiagnosed, which, given the aforementioned (and described in the literature) severe consequences for their current and future health, should be taken as a critical warning for healthcare professionals. Detections and reports are fundamental for early treatment, aiming to avoid an escalation of damage and prevent re-victimization.

## 1 Introduction

The World Health Organization (WHO) describes violence against children as any form of violence that targets people under 18 years of age, perpetrated by someone they know or a stranger (1). The concept of adverse childhood experiences (ACEs) is much broader and consists of potentially traumatic life events that occur in childhood (0–17 years) (2, 3). In the 90s, Felitti et al. enumerated various life events conceivable as ACEs, such as abuse (psychological, physical, and sexual), neglect (psychological and physical), and household dysfunction (exposure to domestic violence, parental separation, substance abuse, mental illness, incarcerated relative, and divorce) (4). Yet, other experiences apply, such as traumatic losses (e.g., by suicide or suicide attempt of someone close) or violence in the community (2). Others may add to the list, such as terrorism, war, torture, living in refugee camps, extreme poverty and homelessness (2, 4, 5).

ACEs are not exclusive to specific households, social strata or settings. Home, school, foster care institutions, and the internet are a few examples of where violence may emerge and impact children (6). Perpetrators may range from family members, neighbors, or peers to perfect strangers or unfamiliar individuals (6, 7).

Despite the rising concern about this issue over the last few years, with an ever-growing body of literature, climaxing in an expectation of a more conscious society, ACEs remain vastly underreported by children’s families, by professionals who work with children (e.g., healthcare, education), and the general community. Frequently, victims are too young to acknowledge some types of abuse or too vulnerable to disclose about them. For instance, corporal punishment masked as a form of child education is still socially accepted in several regions around the world (8) and emotional neglect is not always perceived as abusive.

Globally, it is estimated that half the children aged 2 to 17 years experience physical, emotional, or sexual violence or neglect every year (9). The WHO reports that 300 million 2 to 4 year olds are victims of corporal punishment or psychological violence (or both), perpetrated by parents or other caregivers (10). This entity also declares that one in 5 women and one in 13 men stated having been sexually abused before turning 18 (10).

Infancy and adolescence are exceptionally relevant chapters for developing a healthy foundation from which each person builds their life. This places children in a vulnerable position when faced with adverse experiences and environmental risk factors as it will shape their future as human beings.

ACEs are a worldwide social problem with short-, medium-, and long-term health consequences and ripple effects on children’s lives. During these crucial years, ACEs can result in trauma (11, 12) and intoxications (13–15), mental health disorders (16–19), physical disorders (20–23), and even epigenetic changes (24) as short-term outcomes that may reflect on their adult life. Moreover, worst-case scenarios can result in death (8).

These outcomes have a cumulative effect, increasing with the severity and one’s simultaneous experience of multiple types of violence (4, 25). In their ACE study, Felitti et al. described that exposure to four or more ACEs increased the risk of several mental health and physical disorders (4), which was supported by results of subsequent studies (17, 20, 23, 26).

Healthcare professionals hold a privileged position regarding the ability to identify suspected victims and report the cases to authorities. When violence occurs, victims often are taken to emergency rooms, even if they or the accompanying person do not disclose the reason behind the injuries. Yet, although research conducted in a similar geographical area to ours reported a prevalence of 42.8% of physical abuse at school and 18.5% at home, based on answers to self-administered questionnaires (27) and the WHO reports a 22.9% rate of children victims of physical abuse in Europe (28), a study revealed that 0.6% of occurrences in Portuguese pediatric emergency rooms are seen to derive from violence (29).

Not only do emergency rooms play an indispensable role in this matter, but also primary healthcare, where physicians hold the opportunity to develop a unique doctor-patient dynamic and assess more insidious and recurrent suspected violence situations.

This study aims to analyze the prevalence of health outcomes in children under 16 years of age who were suspected of ACEs by physicians at a local healthcare unit in Portugal. The specific objectives are to describe different health outcomes: (a) traumatic injuries and intoxications; (b) mental health disorders; and (c) physical disorders.

## 2 Materials and methods

### 2.1 Study design and setting

The present study analyzed complementary data from electronic health records and healthcare registries generated during patient interviews. It followed a real-world, retrospective, observational, cross-sectional, and multicentric study approach. Data was extracted from a Portuguese local healthcare unit (LHUM), currently tending approximately 172,557 individuals (29), 40,536 (23.5%) of which are aged 15 years or younger. Located in the North of Portugal, it comprises one hospital and 14 primary healthcare units (12 are family healthcare units and 2 are personalized healthcare units). The option for a local healthcare unit relied on the fact that it includes the variety of services most sought by victims for healthcare purposes. The LHUM is the oldest of the eight Portuguese local healthcare units, matches the geographical area of work of the research team, and has an available and well-cataloged database.

Regarding the eligible population’s size, informed consent is not achievable, therefore we allude to subparagraphs (i) and (j) of Article 9th of the European Union’s General Data Protection Regulation 2016/679 as strong grounds for this study.

Data access was approved by the LHUM Health Ethics Committee and Local Information Protection and Security Committee through approval codes No. 91/CES/JAS on October 14, 2022 (original) and No. 71/CLPSI/2022 on December 21, 2022 (original). The data collection was performed by the Information Technology Department of LHUM and processed and analyzed by analytic programs created solely to this end. This collection was done on LHUM servers, dispensing extracting information outside the LHUM or direct access from researchers. Moreover, the LHUM Information Technology Department de-identified all processed data before the analytic code execution to meet the Health Insurance Portability and Accountability Act (HIPAA) safe harbor criteria. The last data lock point was February 26, 2023.

### 2.2 Participants

Children were included if they fulfilled the four following criteria: (a) to be part of the population served by LHUM; (b) to have had at least one appointment at a LHUM primary healthcare unit; (c) to be suspected of being a victim of violence by a LHUM clinician; (d) to be aged under 16 years old at the time of the event; we chose this cut-off age since other kinds of violence are likely to arise after it, namely dating violence, which was already investigated in another similar study (25).

### 2.3 Variables

We studied the subsequent variables: (a) sex; (b) age; (c) social conditions; (d) traumatic injuries and intoxications; (e) mental health disorders; and (f) physical disorders.

To accomplish that, by listing words and expressions commonly used to describe these events, we came up with a set of keywords and categorized them using the International Classification of Diseases (ICD-9 and ICD-10) and the International Classification of Primary Care (ICPC-2).

All data for the current study purposes were drawn from codes and clinical notes in electronic registries. All categorical variables herein addressed are described in the results, except for those not included due to insufficient data (e.g., sleep disorders, sexually transmitted infections, pregnancy, or hypertension).

### 2.4 Data sources/measurements

Electronic health records and healthcare registries at LHUM were the sources of all data collected from January 1, 2001, to December 31, 2021. Everyone who met the inclusion criteria was enrolled, and no sample was drawn since records from all eligible patients were analyzed.

### 2.5 Bias

Children and/or accompanying people often do not disclose they are being subjected to violence due to not being able to or not recognizing maltreatment. Addition-ally, not all physicians describe or code suspected cases of violence in electronic health records and healthcare registries. Therefore, omission bias represents a real risk in this study given that there might be a prevalence underestimation.

To minimize this bias, we applied broad inclusion criteria, no exclusion criteria, and used the aforementioned set of keywords relevant to this context and all pertinent ICPC-2, ICD-9, and ICD-10 codes.

No other potential sources of bias were identified by the authors.

### 2.6 Statistical methods

We opted for a descriptive analysis of the results, reporting absolute and relative frequencies for all variables. No inferential methods were used.

## 3 Results

Physicians registered a suspicion of ACEs in 2.3% of cases (*n* = 918) from a total population of 40,536 children. Most suspected victims were male (*n* = 552; 60.1%) and the mean age was 9 years (respectively, 52.5% and 8 years for the total population).

The number of suspected victims tends to increase with age, as seen in Supplementary Table 1, in which the last two groups (aged 10–15 years) represent 47% of all victims. There is a reference to unspecified social problems in 1.5% (*n* = 14) of cases, contrasting with 0.4% (*n* = 142) within the total population.

On Supplementary Table 2, the children’s history of traumatic injuries and intoxications, mental health disorders, and psychotropic medication use, as well as some physical disorders are depicted.

## 4 Discussion

Our study assessed existing health problems in children whom physicians had a suspicion of being victims of ACEs. The cases in which a suspicion was registered in an available document account for 2.3% (*n* = 918) of all children. Traumatic injuries, intoxications, as well as mental health and physical disorders were more prevalent within that group of children than in the total studied population (4, 26).

The physiological mechanism underlying this phenomenon is likely to be linked to the chronic stress caused by ACEs, which leads to maladaptive responses to stressful stimuli that end up causing an imbalance of the hypothalamic–pituitary–adrenal axis, as well as neuro-endocrine-immune network disruptions (30, 31). This is associated with higher rates of diseases related to inflammation and hormonal dysfunctions, and with changes in the architecture of the brain, with fewer synapses and dysfunction of certain areas (e.g., prefrontal cortex, amygdala, and hippocampus) that are essential for specific functions like cognition and behavior (32, 33). Furthermore, some epigenetic modifications may arise in children due to a modification in DNA methylation (24) and telomere shortening (34), among other changes.

Nonetheless, such rates depend on the professional’s ability to detect signs and symptoms of childhood adversities that are prevalent yet not as explicit as physical abuse (e.g., neglect, financial hardships, family dysfunction) (35, 36).

### 4.1 Demographics

The total population was composed of similar percentages of female and male children. However, males formed 60.1% of the suspected victims, which leads us to think that they are more frequently abused. Indeed, corporal punishment is one of the most prevalent ACEs in Portugal and, overall, boys tend to have higher odds of being punished (27, 37).

Almost half (47%) of the suspected victims were aged between 10 and 15 years (Supplementary Table 1), which coincides with another Portuguese study in a pediatric emergency setting (29).

Social adversities include financial hardship, lack of access to water, food, transportation, or education, poor habitational conditions, sociocultural adversities, and the death of family members, among others (38). Some of these are intrinsically associated with poverty, a problem that has been described as increasing an individual’s odds of becoming a victim of ACEs (38, 39). Our results showed that 1.5% of the suspected victims had unspecified social problems, which is 3.8 times higher than in the total population. It is probable that some cases were incorrectly coded or described in the clinical notes and that they would otherwise have fallen under the group of “specific social problem.” Regardless, it is worth noting that ACEs affect people of all socioeconomic groups (40) even though there might be less visibility for victims from higher-income families that tend do use private health facilities.

### 4.2 Traumatic injuries and intoxications

When there is a suspicion of abuse, the differential diagnosis regarding the case’s medicolegal etiology may be (41): (a) traumatism (accidental or inflicted—by a third person, self-inflicted or iatrogenic); (b) pathology; or (c) morphological condition.

Most traumatic injuries in ambulatory children are accidental (28). However, physicians need to keep their eyes open to the possibility of an abusive nature and the many possible abuse indicators, such as (41): (a) the injury is not explained by the anamnesis (mechanism and time of wound occurrence); (b) uncommon location for an accidental injury; (c) patterned injuries; (d) symmetric injuries; (e) multiple injuries at different stages of evolution; and (f) delayed search for health care.

Abusive injuries often manifest as skin injuries, with bruises as the most common, affecting up to 90% of physical abuse victims (11) and physical abuse represents 12–20% of all fractures in young children (12). However, our findings showed that superficial injuries were observed in only 3.6% of suspected victims, which is lower when compared to rates for open wounds, bone fractures, and dislocations (see Supplementary Table 2). This may occur because physicians prioritize severe injuries, coding and describing them more thoroughly, over minor or less explicit ones. Additionally, it is noteworthy that children with a history of ACEs are at higher risk of intoxication (14) and self-inflicted injuries (15).

During our study, we found that bone fractures, dislocations, open wounds, superficial injuries, and intoxications were more frequently observed in suspected cases of ACEs compared to the overall population (1.5, 1.9, 1.7, 2.1, and 1.7 times higher, respectively) as depicted in Supplementary Table 2. This suggests that many of these injuries may have been intentional rather than accidental. However, based on our results, we are unable to confirm the medicolegal etiology of these outcomes, which prevents us from making further progress in this regard.

### 4.3 Mental health disorders

The following findings have been reported regarding the prevalence of some challenging behaviors and disorders in children who have experienced ACEs. Both externalizing and internalizing behaviors (42, 43), attention deficit and hyperactivity disorder (ADHD) (43) and the use of psychotropic medication (44) appear to be more prevalent in children with ACEs than in the general population. Another study did an extensive review and found a significant association between sexual abuse and a lifetime diagnosis of anxiety disorder, depression, eating disorders, posttraumatic stress disorder, sleep disorders, and suicide attempts (17). Depression and anxiety symptomatology have also been linked to ACEs based on a similar study population (26).

Our research revealed that higher percentages of suspected ACE victims are associated with mental health disorders and psychotropic medication use, particularly for ADHD and antipsychotic consumption, with rates 10.7 and 7.6 times higher, respectively, compared to the general population—see Supplementary Table 2.

Although data for major depressive disorder were insufficient, suspected victims were found to use antidepressants 5.8 times more frequently than the total population. These disparities may be due to underreporting and the fact that antidepressants are prescribed for indications other than major depressive disorder. It also appears that physicians tend to classify diagnoses broadly as major psychiatric disorders or psychosocial stress, which may encompass many different cases of depressive and anxiety disorders.

### 4.4 Physical disorders

The results of our study displayed type-2 diabetes and hypercholesterolemia as the two most prominent physical disorders among the suspected victims compared to the total population, being 2.9 and 2.1 times higher, respectively (see Supplementary Table 2). Other conditions also evinced higher rates, consistent with several other studies that demonstrated a relationship between ACEs and diabetes (4, 20, 26, 45), cancer (4, 20, 22, 26), hypercholesterolemia (26), asthma (46), and urinary tract infections (47).

In the case of obesity, specifically, which has also been linked to ACEs (4, 20, 35), a study has investigated the role of food addiction in the relationship between childhood trauma and obesity (48). It was suggested that it might potentially account for up to 50% of cases, particularly when overeating becomes a coping mechanism. However, in our study, obesity rates in the total and suspected populations are very similar. This may be due to a significant prevalence of obesity in Portuguese children (15.3% in 2008 and 11.9% in 2019) (49).

These studies also alert to other physical disorders, such as hypertension (26) and stroke (4, 20, 26). We did not include them because the available information was insufficient to allow solid conclusions (hypertension, *n* = 1; stroke, *n* = 3).

### 4.5 The role of healthcare professionals regarding ACEs suspicion

The Portuguese healthcare system is designed to provide the best possible by implementing electronic health records, healthcare registries, and coding systems. Local healthcare units offer a wide range of services, including emergency rooms and primary health care, seeking to increase the likelihood of patients receiving the care they need.

These services are typically free of charge to reduce access barriers for those with fewer financial resources. Healthcare professionals, considering their education and training, are expected to hold the required specific knowledge to identify victims, especially those in close contact with children, which is of paramount importance for the early detection of abuse, neglect and other adversities.

For physical abuse alone, the global known rates are 22.6% (7) and 22.9% in Europe (50). However, the current study found only 2.3% of children assisted at LHUM with suspected ACEs registered by physicians. It is essential to understand why healthcare professionals are not succeeding in suspecting abuse, recording their suspicions, and reporting them.

This unsuccess may be rooted in the following possible reasons: (a) lack of knowledge about indicators of maltreatment and about differentiating them from accidents, self-inflicted injuries, iatrogenic injuries, specific pathological lesions, and other conditions, (b) unawareness of their legal duty to report child abuse or neglect as it is a public crime, (c) unawareness of the impact of abuse on the child’s safety and health, (d) insufficient training on how to report, (e) fear of making an erroneous diagnosis, (f) fear of involvement with the justice system, (g) fear of compromising the doctor-patient trust-based relationship and breaking medical confidentiality duties.

It is crucial not to overlook that these children are at a heightened risk of being abused again (24, 43) and self-harming (12, 13, 15, 16), as well as increasingly developing health problems (15, 47, 48) and dying prematurely (20, 51, 52). The absence of health records and reports from the health system to child protection services or legal authorities will delay or even impede access of the child victim to the support systems (medical, psychological, social, security, and justice-related).

Reporting child abuse cases is mandatory for government employees and physicians according to the Law for the Protection of Children and Adolescents in Danger (Law No. 147/99, September 1, updated version - Law No. 26/2018, 05/07), criminal law (Article 242° of the Portuguese Penal Procedure Code), and the Medical Deontology Regulation of the Portuguese Medical Association (DR, *2ª série*, *n° 139*, 2016). Following the report, the public prosecutor’s office will initiate criminal proceedings as child maltreatment has been considered a public crime since 2001.

### 4.6 Study limitations and further research

We consider that this study’s limitations are: (a) omission bias, possibly caused by the victim’s inability or fear of disclosure, by family’s secrecy (although already anticipated) and by the under-detection of less explicit signs of abuse, neglect or other adversities by physicians (even though we anticipate that some suspicions may have been written as confidential clinical information); (b) absence of an assessment of the number of ACEs per each person, which would have provided an important person-based point of view, considering the dose–response relationship between ACEs and health outcomes (yet this information was not available); (c) absence of health risk behaviors assessment as ACEs outcomes (e.g., substance consumption), since there was not enough data (likely due to a misinterpretation of medical confidentiality); and (d) impossibility to determine the medico-legal etiology of traumatic injuries and intoxications, impeding us from concluding whether or not they had a violent source.

Future research should include other Portuguese healthcare units and European research groups, given it would be pertinent to allow more rock-solid conclusions.

## 5 Conclusion

The following conclusions may be drawn from our study:

(1) Children with ACEs were rated at 2.3% of the total population of child patients (*n* = 40,536). This raises concern for the probable concealment of some abuse or neglect cases by the child or the accompanying person, as well as for the underdetection or underreports by physicians;
(2) Children identified as having ACEs are found to have more health challenges than the rest of the population, with a varying yet seemingly high likelihood:
  a) Traumatic injuries, namely bone fractures, open wounds, bone dislocations, and superficial injuries, are, respectively, 1.5, 1.7, 1.9, 2.1, and 1.7 times higher;
  b) Intoxications, 1.7 times higher;
  c) Major psychiatric disorders, ADHD, social deprivation, psychosocial stress, and unspecified disorders with onset in childhood and adolescence, sum to 5.1, 10.7, 4.2, 1.6, and 21-times higher likelihoods, associated with psychotropics consumption (anxiolytics, antipsychotics, sedatives, and antidepressants, respectively 1.8, 7.6, 4.7, and 5.6 times higher);
  d) Somatic disorders such as metabolic syndrome, obesity, type-2 diabetes, hypercholesterolemia, asthma, urinary tract infections, cancer, and unspecified illnesses, summing up to 1.6, 1.1, 2.9, 2.1, 1.9, 1.9, 2, 1.8 times higher prevalence.

This scenario may be as overwhelming as it may be stimulating if it moves us toward improvement of child victims’ advocacy in healthcare. Laying a strong foundation for children to thrive can be achieved by acting on key areas: (a) promoting positive childhood experiences, with trustworthy adults, and empowering children with age-appropriate information about body safety and other important issues at school, kindergarten and healthcare; (b) advocating for social norms that protect children from adversity and promote resilience by providing systemic support to families; (c) ensuring more training and awareness for professionals working with children, especially in healthcare settings, toward a better detection and report of suspected cases; and (d) establishing and benchmarking a comprehensive national intervention system for suspected cases in countries that lack holistic approaches.

## Data Availability

The original contributions presented in the study are included in the article/Supplementary material, further inquiries can be directed to the corresponding author.
