# Supplementary material for: Adverse childhood experiences and health outcomes: a 20-year real-world study
Source: Front Med (Lausanne). 2025 Jan 7;11:1429137. doi: 10.3389/fmed.2024.1429137 (PMC11745883; doi:10.3389/fmed.2024.1429137)
Supplement: Supplementary file 1 [file Table_1.DOCX]

**Table 1.** Total population and suspected victims of ACEs’ age distribution.

| **AGE GROUPS**  (years) | **Total population**  n (%) | **Suspected ACEs**  n (%) |
| --- | --- | --- |
| 0-3 | 7 264 (17.9) | 160 (17.4) |
| 4-6 | 8 598 (21.2) | 165 (18.0) |
| 7-9 | 7 813 (19.3) | 162 (17.7) |
| 10-12 | 8 627 (21.3) | 201 (21.9) |
| 13-15 | 8 234 (20.3) | 230 (25.1) |
| Total | 40 536 (100.0) | 918 (2.3) |

**Table 2.** Health disorders, injuries and intoxications

|  |  | | **Total population**  n (%) | **Suspected ACEs**  n (%) | **Ratio**  (suspected/total) |
| --- | --- | --- | --- | --- | --- |
| **Mental health disorders** | Major psychiatric disorder | | 1 360 (3.4) | 161 (17.5) | 5.1 |
|  | Attention deficit hyperactivity disorder | | 106 (0.3) | 29 (3.2 | 10.7 |
|  | Social deprivation | | 185 (0.5) | 19 (2.1) | 4.2 |
|  | Psychosocial stress | | 308 (0.8) | 19 (2.1) | 1.6 |
|  | Unspecified disorders with onset in childhood and adolescence | | 52 (0.1) | 19 (2.1) | 21 |
|  | Medication | Anxiolytics | 3 134 (7.7) | 128 (13.9) | 1.8 |
|  |  | Antipsychotics | 414 (1.0) | 70 (7.6) | 7.6 |
|  |  | Sedatives | 432 (1.1) | 48 (5.2) | 4.7 |
|  |  | Antidepressants | 183 (0.5) | 26 (2.8) | 5.6 |
| **Physical disorders** | Metabolic syndrome | | 3 086 (7.6) | 110 (12.0) | 1.6 |
|  | Obesity | | 2 512 (6.2) | 62 (6.8) | 1.1 |
|  | Type 2 diabetes | | 569 (1.4) | 37 (4.0) | 2.9 |
|  | Hypercholesterolemia | | 656 (1.6)) | 30 (3.3) | 2.1 |
|  | Asthma | | 1 189 (2.9) | 51 (5.6) | 1.9 |
|  | Urinary tract infection | | 431 (1.1) | 19 (2.1) | 1.9 |
|  | Cancer | | 252 (0.6) | 11 (1.2) | 2 |
|  | Unspecified illness | | 265 (0.6) | 10 (1.1) | 1.8 |
| **Traumatic injuries**  **and intoxications** | Bone fracture | | 6 652 (16.4) | 218 (23.8) | 1.5 |
|  | Open wound | | 2 260 (5.6) | 88 (9.6) | 1.7 |
|  | Bone dislocation | | 1 034 (2.6) | 46 (5.0) | 1.9 |
|  | Superficial injury | | 688 (1.7) | 33 (3.6) | 2.1 |
|  | Intoxications | | 1 192 (2.9) | 46 (5.0) | 1.7 |
